# Association Between X/Twitter and Prescribing Behavior During the COVID-19 Pandemic: Retrospective Ecological Study

**DOI:** 10.2196/56675

**Published:** 2024-11-18

**Authors:** Scott A Helgeson, Rohan M Mudgalkar, Keith A Jacobs, Augustine S Lee, Devang Sanghavi, Pablo Moreno Franco, Ian S Brooks, National COVID Cohort Collaborative (N3C)

**Affiliations:** 1 Department of Pulmonary and Critical Care Medicine Mayo Clinic Jacksonville, FL United States; 2 School of Information Sciences Center for Health Informatics University of Illinois at Urbana-Champaign Ubana-Champaign, IL United States; 3 Department of Critical Care Medicine Mayo Clinic Jacksonville, FL United States; 4 Department of Transplant Medicine Mayo Clinic Jacksonville, FL United States; 5 see Acknowledgments

**Keywords:** social media, infodemic, COVID-19, healthcare utilization, misinformation, disinformation, Twitter, hydroxychloroquine, X, drugs, pharmacy, pharmacology, pharmacotherapy, pharmaceuticals, medication, prescription, sentiment, SARS-CoV-2, pandemic, respiratory, infectious

## Abstract

**Background:**

Social media has become a vital tool for health care providers to quickly share information. However, its lack of content curation and expertise poses risks of misinformation and premature dissemination of unvalidated data, potentially leading to widespread harmful effects due to the rapid and large-scale spread of incorrect information.

**Objective:**

We aim to determine whether social media had an undue association with the prescribing behavior of hydroxychloroquine, using the COVID-19 pandemic as the setting.

**Methods:**

In this retrospective study, we gathered the use of hydroxychloroquine in 48 hospitals in the United States between January and December 2020. Social media data from X/Twitter was collected using Brandwatch, a commercial aggregator with access to X/Twitter’s data, and focused on mentions of “hydroxychloroquine” and “Plaquenil.” Tweets were categorized by sentiment (positive, negative, or neutral) using Brandwatch’s sentiment analysis tool, with results classified by date. Hydroxychloroquine prescription data from the National COVID Cohort Collaborative for 2020 was used. Granger causality and linear regression models were used to examine relationships between X/Twitter mentions and prescription trends, using optimum time lags determined via vector auto-regression.

**Results:**

A total of 581,748 patients with confirmed COVID-19 were identified. The median daily number of positive COVID-19 cases was 1318.5 (IQR 1005.75-1940.3). Before the first confirmed COVID-19 case, hydroxychloroquine was prescribed at a median rate of 559 (IQR 339.25-728.25) new prescriptions per day. A day-of-the-week effect was noted in both prescriptions and case counts. During the pandemic in 2020, hydroxychloroquine prescriptions increased significantly, with a median of 685.5 (IQR 459.75-897.25) per day, representing a 22.6% rise from baseline. The peak occurred on April 2, 2020, with 3411 prescriptions, a 397.6% increase. Hydroxychloroquine mentions on X/Twitter peaked at 254,770 per day on April 5, 2020, compared to a baseline of 9124 mentions per day before January 21, 2020. During this study’s period, 3,823,595 total tweets were recorded, with 10.09% (n=386,115) positive, 37.87% (n=1,448,030) negative, and 52.03% (n=1,989,450) neutral sentiments. A 1-day lag was identified as the optimal time for causal association between tweets and hydroxychloroquine prescriptions. Univariate analysis showed significant associations across all sentiment types, with the largest impact from positive tweets. Multivariate analysis revealed only neutral and negative tweets significantly affected next-day prescription rates.

**Conclusions:**

During the first year of the COVID-19 pandemic, there was a significant association between X/Twitter mentions and the number of prescriptions of hydroxychloroquine. This study showed that X/Twitter has an association with the prescribing behavior of hydroxychloroquine. Clinicians need to be vigilant about their potential unconscious exposure to social media as a source of medical knowledge, and health systems and organizations need to be more diligent in identifying expertise, source, and quality of evidence when shared on social media platforms.

## Introduction

As of September 1, 2024, SARS-CoV-2 has led to 111,820,082 infections and 1,201,061 deaths reported in the United States alone [1]. When the first nontravel case of COVID-19 was reported in the United States on January 20, 2020, clinicians had no significant knowledge of the virus nor the management of the disease resulting from the virus and looked to colleagues from other countries for their experiences [2,3].

Although peer-reviewed observational reports were being published rapidly in record amounts, and clinical trials were ongoing, clinicians also used social media to share and gain knowledge on the care of their patients in hopes of improving their patients’ outcomes [4-6]. Even though social media is increasingly used by health care providers and health care systems, its accuracy and role as a medium for sharing critical information has the potential to be corrupted, leading to the use of unproven, potentially harmful, and costly therapies [7].

Multiple studies have shown misinformation as shown by false tweets was widely available and were even retweeted faster [8-11]. Not only were there concerns about true and false information spreading, but there was regional sentiment variation on X/Twitter throughout the United States [12]. This misinformation was not only spread by nonmedical people but also by physicians from a range of specialties [13]. To combat this infodemic, the World Health Organization established a repository of COVID-19 fact-checking groups that verify COVID-19 claims [14].

We hypothesized that the circumstances of the COVID-19 pandemic (ie, lack of evidence-based medicine, overly stressed health care system, and health care providers) may have magnified the potential for social media to have influenced the care of patients affected by COVID-19. To explore this hypothesis, we aimed to see if we could identify a temporal association between the X/Twitter mentions of hydroxychloroquine and the “sentiment” of these X/Twitter mentions, against the daily use of the medication.

## Methods

### Social Media Data

All social media data was gathered before the rebranding of Twitter to X, so throughout this paper X will be referred to as X/Twitter and posts will be referred to as tweets. The commercial social media aggregator, Brandwatch was leveraged to gather X/Twitter data and perform sentiment analysis [15,16]. Brandwatch is an official partner of X/Twitter allowing better access to X/Twitter data [17]. The accuracy of Brandwatch’s sentiment analysis is around 75%, which is comparable to other sentiment analysis tools [18-20]. In the tool, a query for the drug “hydroxychloroquine” and “Plaquenil” was added, and the date range was set from November 30, 2019, to January 29, 2021. Unlike other tools that rely on a dictionary of “good” and “bad” words for performing sentiment analysis, Brandwatch’s sentiment analysis models are based on a library of hundreds of rules created based on natural language processing and are updated and audited regularly [16]. Brandwatch classifies tweets as positive or negative only if it is confident about the sentiment classification. If the sentiment cannot be accurately determined, such tweets are categorized as neutral. Tweets were reported as neutral, nonneutral, total, positive, and negative sentiment. Nonneutral tweets combined positive and negative tweets, while total tweets summed up neutral and nonneutral tweets. The tweets were grouped by date, and a CSV file was generated, which included a count of each category of tweets for each day.

### COVID-19 and Medication Data

Medication data was obtained from the National COVID Cohort Collaborative (N3C), a national collection of 48 hospitals or data partners with 4.8 million patients [21]. The N3C cohort is comprised of patients diagnosed with COVID-19 by polymerase chain reaction (PCR) and a control group of patients without COVID-19 matched by age, sex, and race at a 2:1 ratio. For this study, the entire cohort from 2020 was used to capture new hydroxychloroquine prescriptions per day either in the inpatient or in the outpatient setting. Restriction to COVID-19 positivity or negativity was not carried out because the total usage of this medication was being assessed and not specifically the treatment of patients who were tested positive for COVID-19, as patients were treated with hydroxychloroquine before their COVID-19 status resulted, and some of these patients were likely to be negative. Data that could identify patients were not included in the data access, such as age, race, and gender, per N3C. New COVID-19 positivity numbers by PCR were collected with the start date being February 1, 2020, as this was the first date in the N3C database of positive tests. The “drug exposure” dataset of the N3C database was specifically queried for hydroxychloroquine, hydroxychloroquine sulfate, Quineprox, and Plaquenil. There were no exclusion criteria.

### Timeline Data

Prepandemic basal rates of hydroxychloroquine prescriptions and tweets were defined as the period prior from January 1, 2020, to the first reported case of COVID-19 in the United States on January 20, 2020. Pandemic rates were determined using the timeframe between January 21, 2020, to December 31, 2020.

The first peer-reviewed publications on hydroxychloroquine for COVID-19, and key sentinel media or public events or announcements were identified, including announcements from the FDA or other government and nongovernment authorities. A timeline illustrating the X/Twitter hits (including the sentiment) was overlayed to highlight impacts with key media events or announcements, and key peer-reviewed publications evaluating the efficacy of hydroxychloroquine on COVID-19.

### Statistical Analysis

Median and IQRs were reported for descriptive statistics. COVID-19 PCR positivity rates were used to determine the relative use (percent) of hydroxychloroquine prescriptions per patient who tested positive for COVID-19. Estimation of the annual number of hydroxychloroquine prescriptions without the pandemic was carried out by using the median over the prepandemic period and multiplying by 365 (days) to come up with a yearly total. This was used to determine the difference in the number of prescriptions for unnecessary prescriptions. A 10-day prescription and the National Average Drug Acquisition Cost in 2021 were used to calculate the cost of a prescription [22,23].

Statistical analysis was performed using a Granger causality test to determine whether a temporal association exists between X/Twitter mentions and hydroxychloroquine prescriptions [24]. For analysis, the tweets were classified into 3 sentiment categories: neutral, nonneutral, and total as Brandwatch sentiment analysis allows for the most accuracy with this approach.

To obtain the optimum time lag length for Granger causality tests, a vector auto-regressive (VAR) model was fitted to the dataset. The VAR model was run for neutral, nonneutral, and total tweets, and the optimum time lag was noted for each test. Using the optimum time lags calculated from the VAR model, the Granger causality test was then used to determine if a causal relationship exists between X/Twitter mentions of hydroxychloroquine and its prescriptions. The chi-square metric was used to verify the significance of the time lag.

As the Granger causality test does not specify the direction of the temporal association, the optimum lag day was used in a linear regression model to determine whether the number of prescriptions of hydroxychloroquine could be predicted by the number of tweets mentioning hydroxychloroquine in the preceding days (offset by the optimum lag day determined by the above VAR analysis). Similar analyses using a multivariate linear regression model, but using the tweet sentiments as covariates (positive, neutral, and negative sentiment tweets) were carried out to identify their independent impact on predicting the number of new hydroxychloroquine prescriptions. A *P* value of less than .05 was considered significant. Granger analysis was performed using Python (version 3.9.7; Python Software Foundation) using the “stats models” package and the linear regression models were performed in BlueSky (version 10.3; BlueSky Statistics).

### Ethical Considerations

No identifiers were used in the collection or analysis of the data, and this study was considered exempt by the Mayo Clinic institutional review board (#21-002787). This study was approved to use a limited dataset (access level 3) by the N3C Data Use Request Committee. All data were deidentified and a waiver of informed consent was approved consistent with social media postings.

## Results

### COVID-19 and Hydroxychloroquine Prescriptions

**Figure 1:**
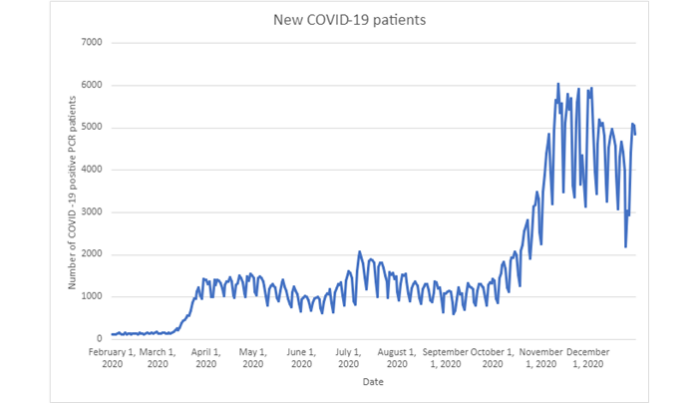
Using the N3C database, the daily census data of new patients with COVID-19 diagnosed through PCR testing was displayed from February 1, 2020, to December 31, 2020. N3C: National COVID Cohort Collaborative; PCR: polymerase chain reaction.

**Figure 2:**
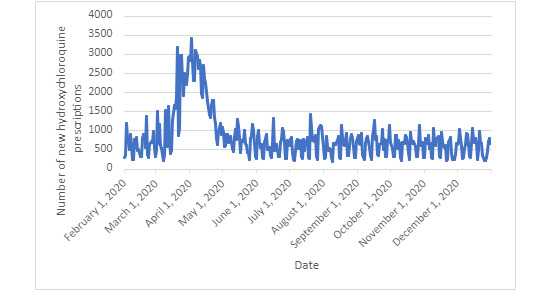
Using the N3C database, daily new hydroxychloroquine prescriptions for any reason were displayed from February 1, 2020, to December 31, 2020. N3C: National COVID Cohort Collaborative.

A total of 581,748 cases of confirmed patients with COVID-19 were identified. The median number of daily positive case counts of patients with COVID-19 was 1318.5 (IQR 1005.75-1940.3) as there was documented reinfection during this study’s period (Figure 1). At a baseline, the median daily use of hydroxychloroquine before the first confirmed case of COVID-19 on January 20, 2020, was 559 (IQR 339.25-728.25) new prescriptions per day (Figure 2). There appears to be a day-of-the-week effect resulting in oscillations of prescriptions and COVID-19 diagnosis. As the pandemic progressed, there was a significant increase in the use of hydroxychloroquine. During the pandemic in 2020, hydroxychloroquine was prescribed a median of 685.5 (IQR 459.75-897.25) times per day, which was a 22.6% increase above the baseline rate. The peak number of prescriptions during a single day was 3411, which occurred on April 2, 2020, which was a 397.6% increase in prescriptions during that day.

### X/Twitter Data

**Figure 3:**
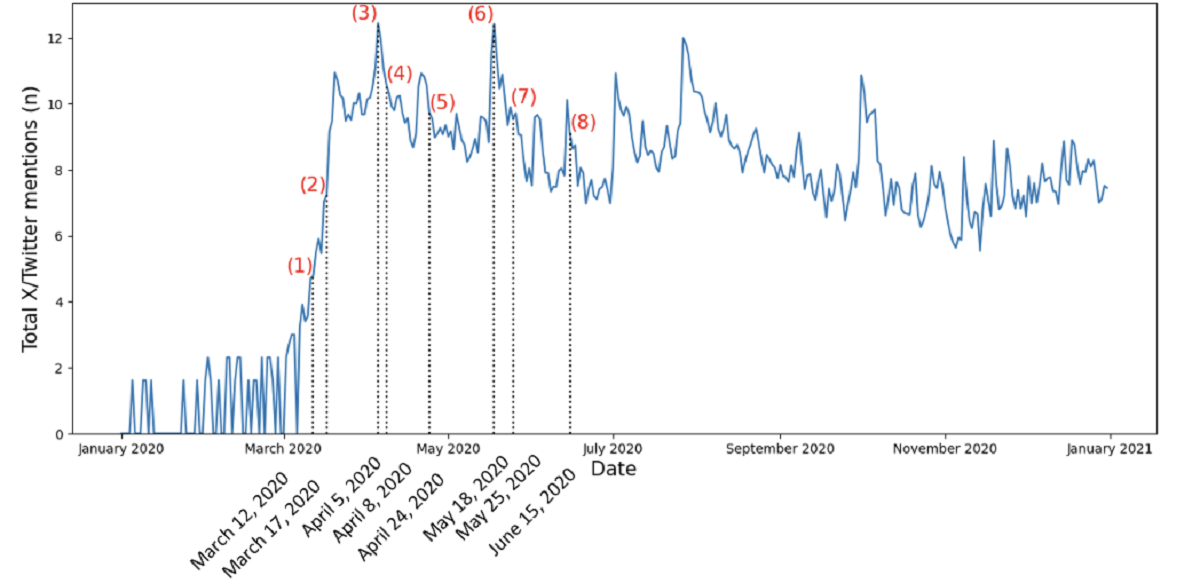
The total X/Twitter mentions of hydroxychloroquine, obtained by Brandwatch, in a natural log scale was displayed from January 1, 2020, to December 31, 2020, and was marked with some key publications and media moments noted by numbers. (1) The first paper was published describing the use of chloroquine for COVID-19 from expert opinion on March 12, 2020 [25]. (2) On March 17, 2020, an open-label nonrandomized study was published showing success with the use of hydroxychloroquine in patients with COVID-19 [26]. (3) On April 5, 2020, the president of the United States promoted the use of hydroxychloroquine for COVID-19 [27]. (4) Then the Center for Disease Control deleted a report of physicians using hydroxychloroquine [3]. (5) The Food and Drug Administration on April 24, 2020, announced to use hydroxychloroquine with caution outside of the hospital or clinical trial secondary to arrhythmias [28]. (6) On May 18, 2020, the president of the United States reported tweets that he is taking hydroxychloroquine to protect himself from COVID-19 [29]. (7) The World Health Organization stopped a trial with hydroxychloroquine over safety concerns [30]. (8) On June 15, 2020, the Food and Drug Administration revoked the Emergency Use Authorization for chloroquine and hydroxychloroquine on COVID-19 [31].

**Figure 4:**
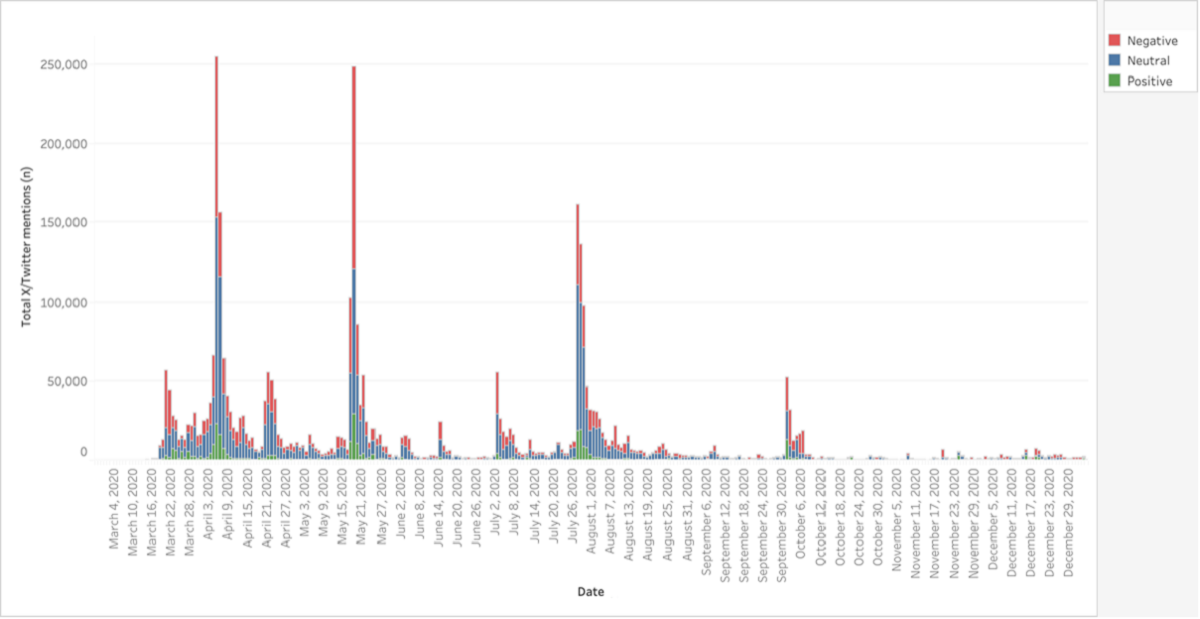
Using Brandwatch, the volume of tweets by total amount and sentiment category (neutral and nonneutral divided out by positive and negative sentiment) was displayed from throughout the year 2020.

Hydroxychloroquine mentions on X/Twitter did not increase significantly until March 2020. X/Twitter mentions and retweets on hydroxychloroquine at baseline were 9124 per day before January 21, 2020. It increased from the pre-COVID baseline and peaked at 254,770 tweets per day on April 5, 2020 (Figure 3 [3,25-31]). There were notable associations between several key publications, public events, or announcements, and a rise in X/Twitter mentions and retweets (Figure 3 [3,25-31]). X/Twitter data were separated into total, neutral, positive, and negative sentiments (Figure 4). For the investigational period, the total tweets mentioning hydroxychloroquine were 3,823,595, with 10.09% (n=386,115) positive, 37.87% (n=1,448,030) negative, and 52.03% (n=1,989,450) neutral sentiments.

### Optimal Lag-Day and Temporal Causality Analysis

The optimal lag-day of 1 day was identified using the VAR model, indicating that the tweets mentioning hydroxychloroquine were most strongly associated with hydroxychloroquine prescriptions within 1 day (Table 1). Although Granger analysis identified significant causal relationships between tweets and prescriptions of hydroxychloroquine across multiple lag days, it confirmed that the strongest statistical relationship occurred with a 1-day time lag identified (total tweets, *P*=.005; neutral tweets, *P*=.001; and nonneutral tweets, *P*=.02; Table S1 in Multimedia Appendix 1).

**Table 1:** Univariate and multivariate analyses for the direction of relationships between tweets and the number of hydroxychloroquine prescriptions with a 1-day time lag through the 2020 year. Tweets were separated by sentiment and total number.

Sentiment		β value	*P* value
**Univariate analysis**			
	Positive	.0641	<.001
	Negative	.0155	<.001
	Neutral	.0169	<.001
	Total	.0078	<.001
**Multivariate analysis**			
	Positive	.0029	.92
	Negative	–.0149	.04
	Neutral	.0271	<.001

### Directional Analysis

The optimal lag day of 1 day demonstrated that an increase in tweets increased hydroxychloroquine prescriptions the following day (Table 1). The biggest impact was with positive tweets, which showed that for every 100 positive sentiment tweets, there would be 6 new hydroxychloroquine prescriptions the next day. Negative tweets, as well as neutral tweets, appeared to be associated with an increase in hydroxychloroquine prescriptions the next day.

However, in multivariate analysis with covariates of positive, negative, and neutral tweets, only neutral and negative tweets seemed to affect hydroxychloroquine prescriptions the next day. On average, the impact of 1000 positive tweets would result in 27.1 new prescriptions the following day, while 1000 negative sentiment tweets would result in 14.9 fewer hydroxychloroquine prescriptions the following day.

### Potential Harm and Costs

In 1 meta-analysis of 9 phase 3 randomized controlled clinical trials, the rate of adverse events with hydroxychloroquine was 12%, with a computed number needed to harm of 9 [32]. The total number of hydroxychloroquine prescriptions over the basal prepandemic period was 68,893. This would translate to 7654.8 patients potentially risking harm if all the hydroxychloroquine prescriptions were for COVID-19. Additionally, hydroxychloroquine National Average Drug Acquisition Costs were estimated at US $3.43 for each new prescription, which may have contributed to an excess of US $236,473.85 in unnecessary costs during this study’s period.

## Discussion

The main findings of our investigation provide temporal evidence that prescribing practices of health care providers were associated with X/Twitter tweets. Specifically, the number of hydroxychloroquine-related tweets was temporally associated with new hydroxychloroquine prescriptions the following day. Supporting the temporal association, multivariate analysis showed that negative sentiment tweets decreased the subsequent prescribing of hydroxychloroquine. Recognizing that hydroxychloroquine has been shown to be ineffective for COVID-19 infection, there may have been 68,893 unnecessary hydroxychloroquine prescriptions resulting in avoidable harm, and US $236,473.85 in excess costs from this study’s sample.

Hydroxychloroquine is an antimalarial medication most often used in the United States to treat rheumatologic disorders. Early reports in the COVID-19 pandemic suggested a potential benefit of hydroxychloroquine in treating COVID-19 [26,32-34]. There was biological plausibility to these studies, with prior in vitro studies showing the activity of hydroxychloroquine against SARS-CoV-1 (severe acute respiratory syndrome coronavirus) and MERS-CoV (Middle East respiratory syndrome) [35,36]. None of the COVID-19 studies reported that considered hydroxychloroquine showed a strong enough level of evidence to meet the criteria for use in patients. The deviation from traditional drug approval pathways and its dissemination to the bedside, which normally takes years, because of the need for effective treatments accelerated this process and arguably led to shortcuts in the process [37]. This scenario led to the backdrop for our study.

The internet era has undoubtedly contributed to improved access and sharing of knowledge and information with physicians and patients [38]. Social media platforms promised to facilitate and strengthen relationships between diverse people and opinions worldwide, by enhancing and supporting collaborations and improving knowledge sharing in the professional world [39,40]. The pace at which information can be shared and disseminated by social media is staggering, considering that previously a confirmed best practice may take more than a decade before its widespread implementation in clinical practice. False news spreads have been reported to spread about 6 times faster and to more than 10 times the people than accurate news when evaluating X/Twitter data [41]. However, much like modern-day centralized electronic health systems, a small error could be magnified and applied systematically across a population of patients unknowingly [42,43]. In the case of social media, there is misinformation (incorrect information) and disinformation (deliberately inaccurate information) on the care of patients that could be magnified, particularly under stressed circumstances, such as a devastating pandemic. X/Twitter responded to this concern by flagging potentially concerning information and removing 11,230 accounts because of misinformation [44]. In the specific case of clinical care, there is concern about circumventing the scientific peer-reviewed process to test the safety of interventions and their risks rigorously. Social media may prematurely propagate preliminary unvalidated therapies such as hydroxychloroquine for COVID-19. Examples of clinical findings conveyed to the media before peer review are suspect and should generally be avoided, with recent examples of disinformation through social media being high doses of vitamin C, thiamine, and hydrocortisone in patients who develop sepsis [45].

COVID-19 caused an infodemic with huge releases of medical literature through peer-reviewed and non–peer-reviewed sources with more than 1000 publications monthly [4]. Along with these publications, technology and social media played a massive role in information dissemination, with millions of tweets weekly about COVID-19 [46]. This sheer volume of data could negatively impact patient care, given that a significant amount of all health- and COVID-19–related tweets contained false information [7]. The World Health Organization raised concerns that this infodemic adversely affected global health related to COVID-19 [47]. Poor-quality health care data have historically been the problem paving the way for a misinformation infodemic [48]. Other prior epidemics (Ebola and Zika) suggested the same infodemic concerns with misinformation spread by social media [49-56]. Outside of epidemics and pandemics, there has also been consistent misinformation about vaccinations portrayed on social media platforms [57-62]. With the COVID-19 pandemic, qualitative studies have shown significant misinformation (up to 70% of information was false or lacked evidence) on social media platforms [7,9,63-67]. One promising study on X/Twitter data showed that false information was tweeted more than science-based information, but science-based tweets were retweeted more [68]. Our study, regarding specifically hydroxychloroquine use, showed that X/Twitter was associated with the prescribing practice during the COVID-19 pandemic, which raises significant concern about a misinformation infodemic.

Some potential fixes to this problem consist of education for physicians or clinicians and appropriate tagging of X/Twitter data. X/Twitter tried to combat misinformation with the implementation of “verified” status, which means that the account is authentic [69]. This verification does not mean that someone is an expert in the field or disease for which they are tweeting. On top of that, the verification system has stopped. Around 39,000 physicians were active on X/Twitter during the start of the pandemic, but it is not known how many provided accurate information. The appropriate use of X/Twitter for health care information or education has not been standardized. There are no educational programs about the appropriate use of X/Twitter and a physician’s responsibility for accurate and verified information. Many of the X/Twitter best practices are geared toward physicians on how to set up their accounts, become noticed, and create connections [70,71]. The development of physician best practices on X/Twitter needs to occur with a focus on disseminating information that is accurate and that nonmedical people will understand. This also creates a need for medical journals to enhance their presence on social media platforms so peer-reviewed studies take the forefront and people can gain access to these studies earlier than for printed copies.

Our findings are compelling but have limitations that should be considered in their interpretation. First, this is an ecological study of multiple databases and their temporal trends, but they are not tied to individual prescribers or patients. For example, we do not know for sure that the use of hydroxychloroquine was prescribed specifically to patients who had COVID-19 and we cannot track whether these prescribing health care professionals used the social media platform. We also do not know if the patients who were prescribed hydroxychloroquine were diagnosed with COVID-19, only had symptoms, or were presumed positive. Additionally, although our findings support that social media did affect COVID-19 prescribing habits, this study did not look at physician versus nonphysician handles. The infodemic could have enhanced the public perception of possible therapies, leading to questioning clinicians taking care of their loved ones, as to why such treatments are being withheld, particularly under the pressure of a patient who is rapidly deteriorating or already on life support [72]. Using X/Twitter as the sample platform to gauge whether social media influenced clinician behavior is faulty, and we acknowledge that social media represents multiple ever-changing platforms and media. However, it is one of the most used social media platforms, with over 396 million users worldwide. The sheer number of hits supported that X/Twitter reasonably represents overall social media sentiments. Sentiment analysis can be difficult because distinguishing between someone being happy that hydroxychloroquine was being used and someone being happy that it was stopped cannot be performed. Brandwatch uses a set of proprietary rules to classify and score tweets into positive, negative, and neutral sentiments. As such, there is no disclosure as to how high these rules classify tweets and we were forced to use a “black box” algorithm, albeit a standard throughout this area of study. Lastly, X/Twitter is not the only source of information and is not the only means of creating undue influence on prescribing habits. There are likely additional factors that cannot be controlled for in this study that impacted prescribing patterns. Additionally, The World Health Organization, Center for Disease Control, hospitals, and medical societies, among others, could have actually mediated the association found in this study.

Similarly, our sample of acute care hospitals in the United States may not precisely reflect the behavior of the rest of the country, as participation and providing data were voluntary, but this is the largest cohort of hospitals to evaluate for prescribing practices [21]. There may be regional differences between academic and nonacademic practices and between types of prescribers, which could not be assessed. We must follow up our findings with more systematic data as they become available (such as from multiple payers and the Center for Medicare Services) and numerous social media platforms. Despite these limitations, our results are compelling, which clinicians, payers, administrators, and policy makers must know as additional supportive data arise.

In conclusion, X/Twitter information about hydroxychloroquine was significantly associated with the prescribing habits of clinicians within 1 day of the tweet. More granular data are necessary to evaluate how specific prescriber details and location affect prescribing practices. The impact of charging for verified X/Twitter accounts may affect future misinformation infodemics, but the primary responsibility for misinformation should be on the person spreading the information and not on the platform or method of dissemination. This study shows a strong analytical case for the dangers of social media and the inappropriate attention and influence on prescribers. Further study is necessary on how to prevent or reduce infodemics in the future as this will likely keep occurring if an intervention is not performed.

## Appendix

Multimedia Appendix 1 Tables on tweet sentiment and tweets per day.

## Abbreviations

MERS-CoV: Middle East respiratory syndrome
N3C: National COVID Cohort Collaborative
PCR: polymerase chain reaction
SARS-CoV-1: severe acute respiratory syndrome coronavirus
VAR: vector auto-regressive

## Data Availability

The prescription and COVID-19 datasets generated and analyzed during this study are not publicly available due to the National COVID Cohort Collaborative data sharing rules, but the X/Twitter data are available from the corresponding author on reasonable request.
