# Supplementary material for: Association Between X/Twitter and Prescribing Behavior During the COVID-19 Pandemic: Retrospective Ecological Study
Source: JMIR Infodemiology. 2024 Nov 18;4:e56675. doi: 10.2196/56675 (PMC11612580; doi:10.2196/56675)
Supplement: Multimedia Appendix 1 [file infodemiology_v4i1e56675_app1.docx]

**Table S1.** Granger causality analysis with different time lags analyzed separated by tweet sentiment.

| **Tweet sentiment** | **Time lag in days** | **p-value** |
| --- | --- | --- |
| Total | 1 | 0.005 |
| Total | 2 | 0.0265 |
| Total | 3 | 0.1603 |
| Total | 4 | 0.3853 |
| Total | 5 | 0.3568 |
| Total | 6 | 0.1637 |
| Total | 7 | 0.0783 |
| Total | 8 | 0.1827 |
| Total | 9 | 0.4827 |
| Total | 10 | 0.522 |
| Neutral | 1 | 0.0012 |
| Neutral | 2 | 0.0049 |
| Neutral | 3 | 0.0383 |
| Neutral | 4 | 0.1299 |
| Neutral | 5 | 0.1519 |
| Neutral | 6 | 0.0741 |
| Neutral | 7 | 0.0341 |
| Neutral | 8 | 0.081 |
| Neutral | 9 | 0.3406 |
| Neutral | 10 | 0.42 |
| Non-neutral | 1 | 0.0239 |
| Non-neutral | 2 | 0.0591 |
| Non-neutral | 3 | 0.3039 |
| Non-neutral | 4 | 0.5828 |
| Non-neutral | 5 | 0.5313 |
| Non-neutral | 6 | 0.2859 |
| Non-neutral | 7 | 0.1327 |
| Non-neutral | 8 | 0.2451 |
| Non-neutral | 9 | 0.5019 |
| Non-neutral | 10 | 0.4815 |

**Table S2.** Percentage of positive, negative, and neutral tweets per day in 2020.

| **Date** | **Positive Tweets Percentage** | **Negative Tweets Percentage** | **Neutral tweets percentage** |
| --- | --- | --- | --- |
| January 1, 2020 | 0 | 0 | 0 |
| January 2, 2020 | 0 | 0 | 0 |
| January 3, 2020 | 0 | 0 | 0 |
| January 4, 2020 | 0 | 0 | 0 |
| January 5, 2020 | 0 | 0 | 100 |
| January 6, 2020 | 0 | 0 | 0 |
| January 7, 2020 | 0 | 0 | 0 |
| January 8, 2020 | 0 | 0 | 0 |
| January 9, 2020 | 0 | 0 | 100 |
| January 10, 2020 | 0 | 0 | 100 |
| January 11, 2020 | 0 | 0 | 0 |
| January 12, 2020 | 100 | 0 | 0 |
| January 13, 2020 | 0 | 0 | 0 |
| January 14, 2020 | 0 | 0 | 0 |
| January 15, 2020 | 0 | 0 | 0 |
| January 16, 2020 | 0 | 0 | 0 |
| January 17, 2020 | 0 | 0 | 0 |
| January 18, 2020 | 0 | 0 | 0 |
| January 19, 2020 | 0 | 0 | 0 |
| January 20, 2020 | 0 | 0 | 0 |
| January 21, 2020 | 0 | 0 | 0 |
| January 22, 2020 | 0 | 0 | 0 |
| January 23, 2020 | 0 | 0 | 0 |
| January 24, 2020 | 0 | 0 | 100 |
| January 25, 2020 | 0 | 0 | 0 |
| January 26, 2020 | 0 | 0 | 0 |
| January 27, 2020 | 0 | 0 | 0 |
| January 28, 2020 | 0 | 0 | 0 |
| January 29, 2020 | 0 | 0 | 100 |
| January 30, 2020 | 0 | 0 | 0 |
| January 31, 2020 | 0 | 0 | 0 |
| February 1, 2020 | 0 | 100 | 0 |
| February 2, 2020 | 100 | 0 | 0 |
| February 3, 2020 | 100 | 0 | 0 |
| February 4, 2020 | 0 | 0 | 0 |
| February 5, 2020 | 0 | 0 | 0 |
| February 6, 2020 | 0 | 0 | 100 |
| February 7, 2020 | 0 | 0 | 0 |
| February 8, 2020 | 0 | 0 | 0 |
| February 9, 2020 | 0 | 0 | 100 |
| February 10, 2020 | 0 | 0 | 100 |
| February 11, 2020 | 0 | 0 | 0 |
| February 12, 2020 | 0 | 0 | 100 |
| February 13, 2020 | 0 | 0 | 100 |
| February 14, 2020 | 0 | 0 | 100 |
| February 15, 2020 | 0 | 0 | 0 |
| February 16, 2020 | 0 | 0 | 0 |
| February 17, 2020 | 100 | 0 | 0 |
| February 18, 2020 | 0 | 0 | 0 |
| February 19, 2020 | 0 | 0 | 100 |
| February 20, 2020 | 0 | 0 | 100 |
| February 21, 2020 | 0 | 0 | 0 |
| February 22, 2020 | 0 | 0 | 100 |
| February 23, 2020 | 0 | 0 | 0 |
| February 24, 2020 | 0 | 50 | 50 |
| February 25, 2020 | 50 | 0 | 50 |
| February 26, 2020 | 0 | 0 | 100 |
| February 27, 2020 | 0 | 0 | 0 |
| February 28, 2020 | 0 | 50 | 50 |
| February 29, 2020 | 0 | 0 | 0 |
| March 1, 2020 | 0 | 0 | 0 |
| March 2, 2020 | 0 | 0 | 100 |
| March 3, 2020 | 33.33 | 33.33 | 33.34 |
| March 4, 2020 | 25 | 0 | 75 |
| March 5, 2020 | 25 | 0 | 75 |
| March 6, 2020 | 0 | 0 | 0 |
| March 7, 2020 | 0 | 60 | 40 |
| March 8, 2020 | 0 | 40 | 60 |
| March 9, 2020 | 0 | 16.67 | 83.33 |
| March 10, 2020 | 0 | 28.57 | 71.43 |
| March 11, 2020 | 9.09 | 31.82 | 59.09 |
| March 12, 2020 | 8.33 | 16.67 | 75 |
| March 13, 2020 | 4.08 | 61.22 | 34.7 |
| March 14, 2020 | 16.44 | 27.4 | 56.16 |
| March 15, 2020 | 10.64 | 19.15 | 70.21 |
| March 16, 2020 | 11.3 | 12.17 | 76.53 |
| March 17, 2020 | 13.41 | 11.59 | 75 |
| March 18, 2020 | 8.86 | 12.56 | 78.58 |
| March 19, 2020 | 9.22 | 39.12 | 51.66 |
| March 20, 2020 | 5.3 | 63.34 | 31.36 |
| March 21, 2020 | 4.57 | 62.63 | 32.8 |
| March 22, 2020 | 26.78 | 26.7 | 46.52 |
| March 23, 2020 | 24.96 | 26.92 | 48.12 |
| March 24, 2020 | 11.07 | 35.03 | 53.9 |
| March 25, 2020 | 35.39 | 13.89 | 50.72 |
| March 26, 2020 | 12.44 | 37.74 | 49.82 |
| March 27, 2020 | 25.16 | 21.3 | 53.54 |
| March 28, 2020 | 11.74 | 43.96 | 44.3 |
| March 29, 2020 | 7.83 | 29.93 | 62.24 |
| March 30, 2020 | 7.08 | 44.18 | 48.74 |
| March 31, 2020 | 15.06 | 39.09 | 45.85 |
| April 1, 2020 | 5.62 | 35.15 | 59.23 |
| April 2, 2020 | 6.68 | 30.69 | 62.63 |
| April 3, 2020 | 12.91 | 36.95 | 50.14 |
| April 4, 2020 | 15.46 | 39.58 | 44.96 |
| April 5, 2020 | 9.24 | 39.87 | 50.89 |
| April 6, 2020 | 10.56 | 25.82 | 63.62 |
| April 7, 2020 | 11.65 | 34.27 | 54.08 |
| April 8, 2020 | 9.16 | 32.18 | 58.66 |
| April 9, 2020 | 7.13 | 38.72 | 54.15 |
| April 10, 2020 | 6.19 | 35.13 | 58.68 |
| April 11, 2020 | 5.3 | 52.57 | 42.13 |
| April 12, 2020 | 4.45 | 57.28 | 38.27 |
| April 13, 2020 | 4.59 | 26.25 | 69.16 |
| April 14, 2020 | 6.16 | 38.67 | 55.17 |
| April 15, 2020 | 9.3 | 37.73 | 52.97 |
| April 16, 2020 | 5.38 | 43.94 | 50.68 |
| April 17, 2020 | 8.62 | 25.02 | 66.36 |
| April 18, 2020 | 30.1 | 18.11 | 51.79 |
| April 19, 2020 | 17.65 | 19.64 | 62.71 |
| April 20, 2020 | 4.08 | 40.71 | 55.21 |
| April 21, 2020 | 5.49 | 35.2 | 59.31 |
| April 22, 2020 | 6.79 | 38.45 | 54.76 |
| April 23, 2020 | 8.18 | 39.86 | 51.96 |
| April 24, 2020 | 7.72 | 47.64 | 44.64 |
| April 25, 2020 | 5.1 | 43.56 | 51.34 |
| April 26, 2020 | 6.1 | 40.88 | 53.02 |
| April 27, 2020 | 9.77 | 18.7 | 71.53 |
| April 28, 2020 | 11.13 | 38.1 | 50.77 |
| April 29, 2020 | 7.31 | 41.89 | 50.8 |
| April 30, 2020 | 5.25 | 15.89 | 78.86 |
| May 1, 2020 | 5.61 | 20.93 | 73.46 |
| May 2, 2020 | 4.06 | 12.95 | 82.99 |
| May 3, 2020 | 8.97 | 47.01 | 44.02 |
| May 4, 2020 | 7.06 | 36.84 | 56.1 |
| May 5, 2020 | 4.49 | 26.03 | 69.48 |
| May 6, 2020 | 5.51 | 26.04 | 68.45 |
| May 7, 2020 | 17.95 | 29.84 | 52.21 |
| May 8, 2020 | 10.17 | 28.53 | 61.3 |
| May 9, 2020 | 6.85 | 26.94 | 66.21 |
| May 10, 2020 | 3.2 | 40 | 56.8 |
| May 11, 2020 | 7.42 | 46.55 | 46.03 |
| May 12, 2020 | 17.54 | 21.61 | 60.85 |
| May 13, 2020 | 8.07 | 50.65 | 41.28 |
| May 14, 2020 | 10.69 | 36.95 | 52.36 |
| May 15, 2020 | 5.51 | 35.39 | 59.1 |
| May 16, 2020 | 5.34 | 45.17 | 49.49 |
| May 17, 2020 | 11.4 | 46.51 | 42.09 |
| May 18, 2020 | 11.96 | 51.15 | 36.89 |
| May 19, 2020 | 12.29 | 37.76 | 49.95 |
| May 20, 2020 | 8.6 | 28.78 | 62.62 |
| May 21, 2020 | 7.77 | 38.28 | 53.95 |
| May 22, 2020 | 5.79 | 35.11 | 59.1 |
| May 23, 2020 | 14.66 | 36.07 | 49.27 |
| May 24, 2020 | 20.38 | 35.45 | 44.17 |
| May 25, 2020 | 5.48 | 33.33 | 61.19 |
| May 26, 2020 | 6.83 | 38.12 | 55.05 |
| May 27, 2020 | 15.99 | 31.24 | 52.77 |
| May 28, 2020 | 5.03 | 50.12 | 44.85 |
| May 29, 2020 | 6.87 | 47.67 | 45.46 |
| May 30, 2020 | 7.69 | 37.5 | 54.81 |
| May 31, 2020 | 4.82 | 20.74 | 74.44 |
| June 1, 2020 | 5.12 | 29.38 | 65.5 |
| June 2, 2020 | 11.63 | 33.14 | 55.23 |
| June 3, 2020 | 4.31 | 45.26 | 50.43 |
| June 4, 2020 | 1.89 | 52.27 | 45.84 |
| June 5, 2020 | 3.82 | 31.3 | 64.88 |
| June 6, 2020 | 4.62 | 50.09 | 45.29 |
| June 7, 2020 | 4.4 | 41.1 | 54.5 |
| June 8, 2020 | 6.51 | 40.72 | 52.77 |
| June 9, 2020 | 6.41 | 49.3 | 44.29 |
| June 10, 2020 | 7.93 | 38.53 | 53.54 |
| June 11, 2020 | 3.94 | 38.87 | 57.19 |
| June 12, 2020 | 4.66 | 68.33 | 27.01 |
| June 13, 2020 | 5.4 | 59 | 35.6 |
| June 14, 2020 | 6.7 | 46.03 | 47.27 |
| June 15, 2020 | 8.09 | 40.93 | 50.98 |
| June 16, 2020 | 7.6 | 36.37 | 56.03 |
| June 17, 2020 | 4.85 | 37.15 | 58 |
| June 18, 2020 | 11.26 | 36.81 | 51.93 |
| June 19, 2020 | 6.97 | 15.21 | 77.82 |
| June 20, 2020 | 4.05 | 14.18 | 81.77 |
| June 21, 2020 | 12.62 | 32.24 | 55.14 |
| June 22, 2020 | 4.8 | 37.84 | 57.36 |
| June 23, 2020 | 5.01 | 63.33 | 31.66 |
| June 24, 2020 | 19.78 | 38.43 | 41.79 |
| June 25, 2020 | 21.63 | 22.04 | 56.33 |
| June 26, 2020 | 10.21 | 64.56 | 25.23 |
| June 27, 2020 | 7.65 | 71.1 | 21.25 |
| June 28, 2020 | 4.17 | 51.75 | 44.08 |
| June 29, 2020 | 8.7 | 32.92 | 58.38 |
| June 30, 2020 | 9.26 | 37.5 | 53.24 |
| July 1, 2020 | 4.15 | 12.6 | 83.25 |
| July 2, 2020 | 8.32 | 46.75 | 44.93 |
| July 3, 2020 | 7.9 | 39.91 | 52.19 |
| July 4, 2020 | 2.13 | 61.83 | 36.04 |
| July 5, 2020 | 2.7 | 37.63 | 59.67 |
| July 6, 2020 | 5.88 | 36.46 | 57.66 |
| July 7, 2020 | 10.51 | 38.99 | 50.5 |
| July 8, 2020 | 8.86 | 47.17 | 43.97 |
| July 9, 2020 | 4.26 | 40.81 | 54.93 |
| July 10, 2020 | 5.11 | 48.86 | 46.03 |
| July 11, 2020 | 36.35 | 37.02 | 26.63 |
| July 12, 2020 | 11.7 | 48.5 | 39.8 |
| July 13, 2020 | 5.36 | 45.02 | 49.62 |
| July 14, 2020 | 4.41 | 36.71 | 58.88 |
| July 15, 2020 | 5.48 | 25.61 | 68.91 |
| July 16, 2020 | 9.61 | 19.84 | 70.55 |
| July 17, 2020 | 4.98 | 43.23 | 51.79 |
| July 18, 2020 | 6.04 | 34.99 | 58.97 |
| July 19, 2020 | 2.76 | 29.36 | 67.88 |
| July 20, 2020 | 5.96 | 20.47 | 73.57 |
| July 21, 2020 | 6.67 | 12.31 | 81.02 |
| July 22, 2020 | 8.75 | 24.76 | 66.49 |
| July 23, 2020 | 7.13 | 24.55 | 68.32 |
| July 24, 2020 | 4.16 | 26.97 | 68.87 |
| July 25, 2020 | 11.38 | 26.52 | 62.1 |
| July 26, 2020 | 15.82 | 33.87 | 50.31 |
| July 27, 2020 | 11.73 | 31.49 | 56.78 |
| July 28, 2020 | 14.3 | 27.29 | 58.41 |
| July 29, 2020 | 8.72 | 27.21 | 64.07 |
| July 30, 2020 | 17.99 | 29.37 | 52.64 |
| July 31, 2020 | 12.57 | 40.4 | 47.03 |
| August 1, 2020 | 5.55 | 33.42 | 61.03 |
| August 2, 2020 | 7.05 | 33.79 | 59.16 |
| August 3, 2020 | 7.88 | 22.26 | 69.86 |
| August 4, 2020 | 6.34 | 18.83 | 74.83 |
| August 5, 2020 | 9.57 | 35.39 | 55.04 |
| August 6, 2020 | 8.87 | 34.47 | 56.66 |
| August 7, 2020 | 3.95 | 28.31 | 67.74 |
| August 8, 2020 | 3.78 | 65.34 | 30.88 |
| August 9, 2020 | 4.35 | 38.34 | 57.31 |
| August 10, 2020 | 8.83 | 29.34 | 61.83 |
| August 11, 2020 | 11.06 | 58.07 | 30.87 |
| August 12, 2020 | 8.47 | 26.9 | 64.63 |
| August 13, 2020 | 5.33 | 31.79 | 62.88 |
| August 14, 2020 | 4.56 | 22.72 | 72.72 |
| August 15, 2020 | 7.97 | 22.14 | 69.89 |
| August 16, 2020 | 5.99 | 34.31 | 59.7 |
| August 17, 2020 | 13.99 | 43.86 | 42.15 |
| August 18, 2020 | 7.54 | 33.46 | 59 |
| August 19, 2020 | 6.54 | 25.9 | 67.56 |
| August 20, 2020 | 3.57 | 14.37 | 82.06 |
| August 21, 2020 | 7.36 | 27.34 | 65.3 |
| August 22, 2020 | 5.71 | 46.06 | 48.23 |
| August 23, 2020 | 8.28 | 30.67 | 61.05 |
| August 24, 2020 | 6.27 | 59.48 | 34.25 |
| August 25, 2020 | 24.64 | 30.98 | 44.38 |
| August 26, 2020 | 11.38 | 23.85 | 64.77 |
| August 27, 2020 | 3.64 | 50.16 | 46.2 |
| August 28, 2020 | 7.72 | 66.18 | 26.1 |
| August 29, 2020 | 7.84 | 37.59 | 54.57 |
| August 30, 2020 | 7.12 | 46.99 | 45.89 |
| August 31, 2020 | 7.59 | 23.43 | 68.98 |
| September 1, 2020 | 4.8 | 19.91 | 75.29 |
| September 2, 2020 | 7.13 | 35.64 | 57.23 |
| September 3, 2020 | 8.58 | 19.67 | 71.75 |
| September 4, 2020 | 8.31 | 24.21 | 67.48 |
| September 5, 2020 | 8.84 | 28.72 | 62.44 |
| September 6, 2020 | 22.55 | 39.04 | 38.41 |
| September 7, 2020 | 7.53 | 23.96 | 68.51 |
| September 8, 2020 | 2.34 | 35.63 | 62.03 |
| September 9, 2020 | 16.76 | 28.03 | 55.21 |
| September 10, 2020 | 13.37 | 23.1 | 63.53 |
| September 11, 2020 | 4.83 | 23.14 | 72.03 |
| September 12, 2020 | 3.25 | 10.33 | 86.42 |
| September 13, 2020 | 15.44 | 28.19 | 56.37 |
| September 14, 2020 | 13.28 | 33.61 | 53.11 |
| September 15, 2020 | 6.67 | 20 | 73.33 |
| September 16, 2020 | 5.32 | 15.78 | 78.9 |
| September 17, 2020 | 7.17 | 56.27 | 36.56 |
| September 18, 2020 | 5.67 | 21.28 | 73.05 |
| September 19, 2020 | 2.37 | 44.21 | 53.42 |
| September 20, 2020 | 6.47 | 36.64 | 56.89 |
| September 21, 2020 | 16.56 | 31.85 | 51.59 |
| September 22, 2020 | 8.69 | 58.55 | 32.76 |
| September 23, 2020 | 2.94 | 53.39 | 43.67 |
| September 24, 2020 | 8.08 | 47.69 | 44.23 |
| September 25, 2020 | 11.81 | 40.94 | 47.25 |
| September 26, 2020 | 8.49 | 34.91 | 56.6 |
| September 27, 2020 | 4.42 | 14.2 | 81.38 |
| September 28, 2020 | 2.7 | 28.06 | 69.24 |
| September 29, 2020 | 5.46 | 15.8 | 78.74 |
| September 30, 2020 | 2.08 | 14.91 | 83.01 |
| October 1, 2020 | 25.13 | 39.16 | 35.71 |
| October 2, 2020 | 5.19 | 71.04 | 23.77 |
| October 3, 2020 | 7.75 | 49.28 | 42.97 |
| October 4, 2020 | 10.33 | 16.58 | 73.09 |
| October 5, 2020 | 2.53 | 74.75 | 22.72 |
| October 6, 2020 | 6.95 | 74.77 | 18.28 |
| October 7, 2020 | 21.97 | 40.49 | 37.54 |
| October 8, 2020 | 7.04 | 32.47 | 60.49 |
| October 9, 2020 | 4.49 | 50.9 | 44.61 |
| October 10, 2020 | 8 | 54 | 38 |
| October 11, 2020 | 16.67 | 47.22 | 36.11 |
| October 12, 2020 | 2.65 | 34.66 | 62.69 |
| October 13, 2020 | 5.39 | 41.18 | 53.43 |
| October 14, 2020 | 2.21 | 24.32 | 73.47 |
| October 15, 2020 | 4.03 | 34.29 | 61.68 |
| October 16, 2020 | 8.99 | 41.57 | 49.44 |
| October 17, 2020 | 1.89 | 27.04 | 71.07 |
| October 18, 2020 | 7.69 | 48.72 | 43.59 |
| October 19, 2020 | 8.44 | 40.91 | 50.65 |
| October 20, 2020 | 33.06 | 11.29 | 55.65 |
| October 21, 2020 | 64.23 | 5.62 | 30.15 |
| October 22, 2020 | 16.56 | 35.1 | 48.34 |
| October 23, 2020 | 18.1 | 24.76 | 57.14 |
| October 24, 2020 | 7.09 | 42.52 | 50.39 |
| October 25, 2020 | 23.12 | 15.61 | 61.27 |
| October 26, 2020 | 9.96 | 17.71 | 72.33 |
| October 27, 2020 | 2.4 | 5.86 | 91.74 |
| October 28, 2020 | 2.04 | 27.04 | 70.92 |
| October 29, 2020 | 2.05 | 53.08 | 44.87 |
| October 30, 2020 | 3.55 | 24.65 | 71.8 |
| October 31, 2020 | 3.55 | 33.06 | 63.39 |
| November 1, 2020 | 10.88 | 21.24 | 67.88 |
| November 2, 2020 | 7.2 | 16 | 76.8 |
| November 3, 2020 | 2.41 | 18.07 | 79.52 |
| November 4, 2020 | 16.92 | 35.38 | 47.7 |
| November 5, 2020 | 8.93 | 32.14 | 58.93 |
| November 6, 2020 | 11.84 | 22.37 | 65.79 |
| November 7, 2020 | 11.27 | 29.58 | 59.15 |
| November 8, 2020 | 4.11 | 23.43 | 72.46 |
| November 9, 2020 | 16.91 | 24.63 | 58.46 |
| November 10, 2020 | 15.87 | 31.75 | 52.38 |
| November 11, 2020 | 8.91 | 32.67 | 58.42 |
| November 12, 2020 | 6.63 | 20.48 | 72.89 |
| November 13, 2020 | 4.55 | 52.6 | 42.85 |
| November 14, 2020 | 9.8 | 47.06 | 43.14 |
| November 15, 2020 | 8.6 | 63.44 | 27.96 |
| November 16, 2020 | 1.3 | 21.24 | 77.46 |
| November 17, 2020 | 7.63 | 27.97 | 64.4 |
| November 18, 2020 | 12.93 | 31.29 | 55.78 |
| November 19, 2020 | 2.99 | 72.2 | 24.81 |
| November 20, 2020 | 7.02 | 43.11 | 49.87 |
| November 21, 2020 | 8.33 | 40 | 51.67 |
| November 22, 2020 | 6.53 | 18.09 | 75.38 |
| November 23, 2020 | 6.23 | 19.78 | 73.99 |
| November 24, 2020 | 51.42 | 11.06 | 37.52 |
| November 25, 2020 | 23.91 | 10.06 | 66.03 |
| November 26, 2020 | 5.26 | 15.79 | 78.95 |
| November 27, 2020 | 4.89 | 32.07 | 63.04 |
| November 28, 2020 | 1.86 | 62.11 | 36.03 |
| November 29, 2020 | 14.13 | 26.09 | 59.78 |
| November 30, 2020 | 1.83 | 14.29 | 83.88 |
| December 1, 2020 | 6.9 | 29.66 | 63.44 |
| December 2, 2020 | 4.32 | 77.57 | 18.11 |
| December 3, 2020 | 4.67 | 62.62 | 32.71 |
| December 4, 2020 | 3.79 | 8.84 | 87.37 |
| December 5, 2020 | 7.69 | 33.48 | 58.83 |
| December 6, 2020 | 6.32 | 41.84 | 51.84 |
| December 7, 2020 | 5.53 | 60.86 | 33.61 |
| December 8, 2020 | 5.3 | 73.25 | 21.45 |
| December 9, 2020 | 5.18 | 70.19 | 24.63 |
| December 10, 2020 | 5.67 | 20.17 | 74.16 |
| December 11, 2020 | 7.37 | 58.65 | 33.98 |
| December 12, 2020 | 6.58 | 76.32 | 17.1 |
| December 13, 2020 | 11.43 | 36.67 | 51.9 |
| December 14, 2020 | 30.8 | 14.72 | 54.48 |
| December 15, 2020 | 41.37 | 30.13 | 28.5 |
| December 16, 2020 | 42 | 25.29 | 32.71 |
| December 17, 2020 | 8.42 | 58.68 | 32.9 |
| December 18, 2020 | 35 | 58.22 | 6.78 |
| December 19, 2020 | 27.88 | 35.55 | 36.57 |
| December 20, 2020 | 11.3 | 17.1 | 71.6 |
| December 21, 2020 | 6.7 | 12.89 | 80.41 |
| December 22, 2020 | 4.53 | 17.42 | 78.05 |
| December 23, 2020 | 4.89 | 57.79 | 37.32 |
| December 24, 2020 | 1.6 | 61.55 | 36.85 |
| December 25, 2020 | 1.69 | 33.64 | 64.67 |
| December 26, 2020 | 1.91 | 44.78 | 53.31 |
| December 27, 2020 | 2.56 | 69 | 28.44 |
| December 28, 2020 | 9.09 | 48.18 | 42.73 |
| December 29, 2020 | 4.05 | 50.61 | 45.34 |
| December 30, 2020 | 5.18 | 81.2 | 13.62 |
| December 31, 2020 | 10.47 | 79.65 | 9.88 |
